# Measuring CO in an anti-smoking campaign in Virovitičko-podravska County in Croatia

**DOI:** 10.1192/j.eurpsy.2024.1407

**Published:** 2024-08-27

**Authors:** S. Mihaljević, M. Beneš, M. Venus

**Affiliations:** Institute of Public Health “Sveti Rok” Virovitičko - podravska County, Virovitica, Croatia

## Abstract

**Introduction:**

Measuring carbon monoxide (CO) in exhaled breath with a visual representation can aid in smoking cessation by increasing smokers’ awareness of how smoking negatively affects their health and how many harmful substances they introduce into their bodies. Individuals attempting to quit smoking can regularly measure CO levels to monitor their progress in reducing this gas in their system.

The national „Smoke Out Day” is a day aimed at encouraging smokers to quit smoking in the Republic of Croatia. It is celebrated on the first day of Lent, as it is a period when most people contemplate giving up something they enjoy throughout the year.

The Institute of Public Health of Virovitičko-podravska County sets up a booth in one of the cities in County on that day. There we offer pamphlets to passersby with information about the harmful effects of smoking and provide them with the opportunity to measure carbon monoxide in their exhaled breath.

**Objectives:**

The aim of this study was to explore relationship between smoking status, smoke exposure and the levels of exhaled carbon monoxide.

**Methods:**

For measuring CO we used piCO simple Smokerlyzer. From April 2015 till May 2023, 462 individuals have participated in this measurement. The data were processed using the Python programming language version 3.9 and the statistical library Pingouin version 0.5.3. Data visualization was performed using the Seaborn library version 0.12.2. The statistical analyses employed in this study are partial correlations. The Spearman method was used for partial correlations because it can identify nonlinear relationships and is more accurate and robust when the assumptions for Pearson correlation are not met.

**Results:**

Partial correlation analysis indicates that there is a statistically significant relationship between the level of carbon monoxide and the following parameters:

- Non-smoking status in years (rho = -0.526, p <0.001, two-tailed test).
- Number of cigarettes smoked per day (rho = 0.369, p <0.001, two-tailed test).
- Testing time after 3 PM (rho = 0.234, p <0.001, two-tailed test).
- Number of years as a smoker (rho = 0.230, p <0.001, two-tailed test).
- Age in years (rho = -0.179, p <0.001, two-tailed test).
- Time spent in a smoky area (rho = 0.114, p <0.016, two-tailed test).

**Conclusions:**

In summary, these results provide valuable insights into the factors associated with carbon monoxide levels in humans, with smoking-related variables, age, and testing time showing notable partial correlations. It is important to consider these relationships when assessing and managing carbon monoxide exposure and its potential health implications.

**Disclosure of Interest:**

None Declared

